# Descriptions of hitherto unknown larvae of the genus *Hydropsyche* Pictet, 1834 from China (Trichoptera, Hydropsychidae)

**DOI:** 10.3897/BDJ.13.e151321

**Published:** 2025-03-17

**Authors:** Xinyu Ge, Jingyuan Wang, Lu Chai, Chuncai Yan

**Affiliations:** 1 Tianjin Key Laboratory of Conservation and Utilization of Animal Diversity, College of Life Sciences, Tianjin Normal University, Tianjin, China Tianjin Key Laboratory of Conservation and Utilization of Animal Diversity, College of Life Sciences, Tianjin Normal University Tianjin China

**Keywords:** taxonomy, Hydropsychinae, identification, DNA barcoding

## Abstract

**Background:**

*Hydropsyche* Pictet, 1834 is the largest genus of Hydropsychinae. In China, larval descriptions exist for only about 20 species. Although the number of *Hydropsyche* larvae described in China has increased rapidly in recent years, larvae of more than 75% of Chinese *Hydropsyche* species remain unknown.

**New information:**

In this paper, we describe and illustrate the larvae of *Hydropsychebriareus* Malicky & Chantaramongkol, 2000 and *Hydropsychekozhantschikovi* Martynov, 1924 for the first time. Neighbour-joining trees were reconstructed, based on known partial *Hydropsyche* species *mtCOI* barcodes.

## Introduction

Trichoptera is one of the most diverse and abundant groups of insects found in various habitats in aquatic ecosystems, widely used to evaluate the impact of aquatic system (Morse et al. 2019, Ge et al. 2024). With more than 2000 described species, Hydropsychidae Curtis 1835 is the largest family in Annulipalpia and is distributed on all continents, except Antarctica (Geraci et al. 2005, Oláh and Johanson 2008). The subfamily Hydropsychinae constitutes an ecologically significant caddisfly group, exhibiting the highest species diversity and biomass amongst Trichoptera in many lotic habitats (Ge et al. 2022c). The genus *Hydropsyche* is the largest genus of subfamily Hydropsychinae. It is represented by 578 species globally and most of these species are confined to the Oriental and Palaearctic Regions (Oláh et al. 2020, Morse 2025). Moreover, their filter-feeding activity provides various important ecosystem services.

The genus *Hydropsyche* has become an increasingly prominent subject of entomological research in recent years, with studies encompassing both larval and adult life stages (Oláh and Johanson 2008, Prommi 2016, Pandher et al. 2017, Karaouzas 2018, Karaouzas and Kapakos 2018, Vicentini et al. 2020). In China, this taxon is currently represented by 86 documented species (Yang et al. 2016, Oláh et al. 2020, Ge et al. 2023). Amongst these, larval descriptions exist for only 20 species (Zhou 2007, Xu et al. 2018, Ge et al. 2020). Although the number of *Hydropsyche* larvae described in China has increased rapidly in recent years, larvae of more than 75% of Chinese *Hydropsyche* species remain unknown. This highlights a significant taxonomic gap in our understanding of this ecologically important group.

Distinguishing between aquatic larval stages remains challenging, as these developmental phases cannot be reliably separated using purely morphological criteria (Rossaro et al. 2022). DNA barcodes corresponding to the 658-bp fragment of the mitochondrial cytochrome *c* oxidase I (*mtCOI*) gene have been established as the foundational component of global species-level bioidentification systems (Hebert et al. 2003). This molecular marker has provided important evidence to confirm new species and has proved to be useful for association between trichopteran larvae and adults (Zhou 2007, Zhang et al. 2021, Ge et al. 2022a, Zang et al. 2022, Peng et al. 2023).

In this study, we describe and illustrate the larvae of *Hydropsychebriareus* Malicky & Chantaramongkol, 2000 and *Hydropsychekozhantschikovi* Martynov, 1924 for the first time. *Hydropsychebriareus* and *H.kozhantschikovi* are distributed in the Oriental Region and Palaearctic Region, respectively. We matched and identified larval stages of these two species, based on DNA barcodes.

## Materials and methods

### Sample collection

Adult and larval specimens were collected using 15-w ultraviolet light tubes and a D-frame aquatic net in Yunnan Province, Liaoning Province, Heilongjian Province etc., PR China during July 2023 — August 2024. Specimens were collected by Xinyu Ge, Wei Cao, Qingbo Huo and Dewen Gong. All specimens were stored in 95% alcohol immediately after collection. Voucher specimens are deposited at the Insect Collection, Tianjin Normal University, Tianjin, PR China.

### Morphological study

Adults and larvae were examined with a stereomicroscope (LEICA M205 C). The identification of 5^th^ instar larvae was based on the presence or absence of pupal gill buds on their abdomens, as described by Vicentini et al. (2020). Adults were determined according to Oláh and Johanson (2008). Photographs of the larvae were captured using a stereomicroscope equipped with a built-in Leica Microsystems CMS GmbH (K5C-BZ01). A series of images was taken at varying focal depths, which were automatically stacked using Leica Application Suite X software (version 5.1.0.25593). Subsequently, these images were arranged and edited in Adobe Photoshop® (version 23.2.1 20220222) to produce final illustrations.

### Terminology

The terminology for overall morphology of larval characters follows that of Wiggins (1996), Zhou (2007) and Xu et al. (2018).

### Molecular analysis

Genomic DNA was extracted from the right hind-leg of adult and larvae specimens, using the animal tissue protocol provided by the TIANamp Genomic DNA Kit. The *mtCOI* barcoding (658-bp) was amplified and analysed using PCR following the protocol by Ge et al. (2022b). The primers of PCR (LCO1490/HCO2198) are listed in Table 1. The *mtCOI* barcoding was sequenced by Beijing Genomics Institute (Beijing) and the raw sequences were manually assembled and edited using Sequencher v.4.5 (Gene Codes Corporation, Ann Arbor, Michigan, USA) and Mega X (Kumar et al. 2018). A neighbour-joining (NJ) tree of 30 sequences of five species within the genus *Hydropsyche* was constructed using Mega X (Suppl. material 1), with the following parameters: Kimura 2-parameter substitution model (K2P) and others as defaults.

## Data resources

The newly-sequenced species in this study have been uploaded to GenBank (PV174544—PV174562).

## Taxon treatments

### Hydropsyche briareus

Malicky & Chantaramongkol, 2000

060ACA3A-F68F-5440-B5E6-ED61612D00C9

#### Materials

- **Type status:** Other material. **Occurrence:** sex: 2 males; lifeStage: 2 adults, 3 larvae; occurrenceID: EBD13007-C109-5C60-AC1B-6985D2E586FF; **Location:** country: China; stateProvince: Sichuan; county: Ningnan; locality: Hulukou Town, Heishui River; verbatimElevation: 610m; verbatimLatitude: 26°57'46"N; verbatimLongitude: 102°48'27"E; verbatimCoordinateSystem: degrees minutes seconds; **Identification:** identifiedBy: Xinyu Ge; **Event:** samplingProtocol: UV light trap, D-frame aquatic net; eventDate: 22 Nov 2023; **Record Level:** institutionCode: Tianjin Normal University, Tianjin, China (TJNU)
- **Type status:** Other material. **Occurrence:** lifeStage: 2 larvae; occurrenceID: 4E6E270B-B296-5096-89AE-7FDF792F9C2F; **Location:** country: China; stateProvince: Yunnan; county: Yuanmou; locality: Jianshe Village, Longchuan River; verbatimElevation: 880m; verbatimLatitude: 25°57'39" N; verbatimLongitude: 101°52'28" E; verbatimCoordinateSystem: degrees minutes seconds; **Identification:** identifiedBy: Xinyu Ge; **Event:** samplingProtocol: D-frame aquatic net; eventDate: 19 Nov 2023; **Record Level:** institutionCode: Tianjin Normal University, Tianjin, China (TJNU)

#### Description

**Figure 1.**
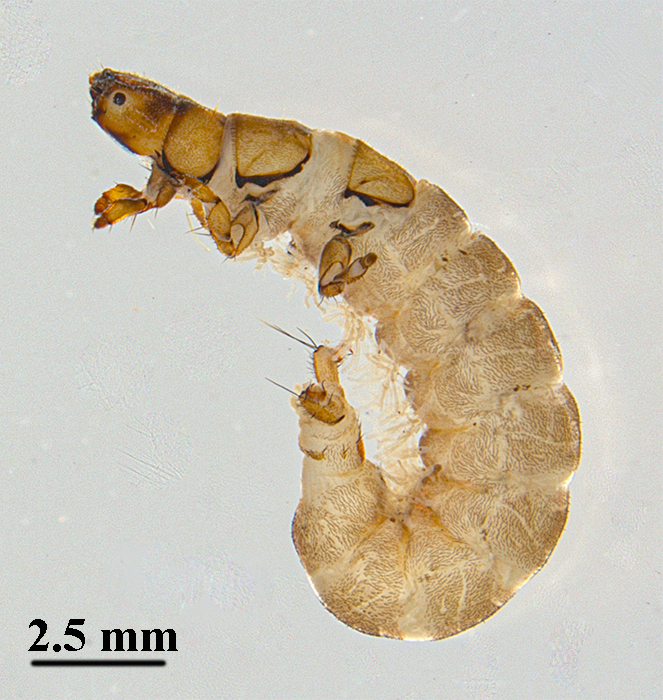
The overall body shape of larva of *H.briareus* in lateral view.

Mean length of 5^th^ instar larvae is about 15 mm (n = 5). Overall body length is similar to most hydropsychid larvae (Fig. 1). The colours of the specimens in alcohol are as follows: body creamy-white; head features a mottled pattern of dark brown and yellowish-brown; pronotum, ventral prosternum, abdominal sternum, legs and anal claws yellowish-brown.

**Figure 2.**
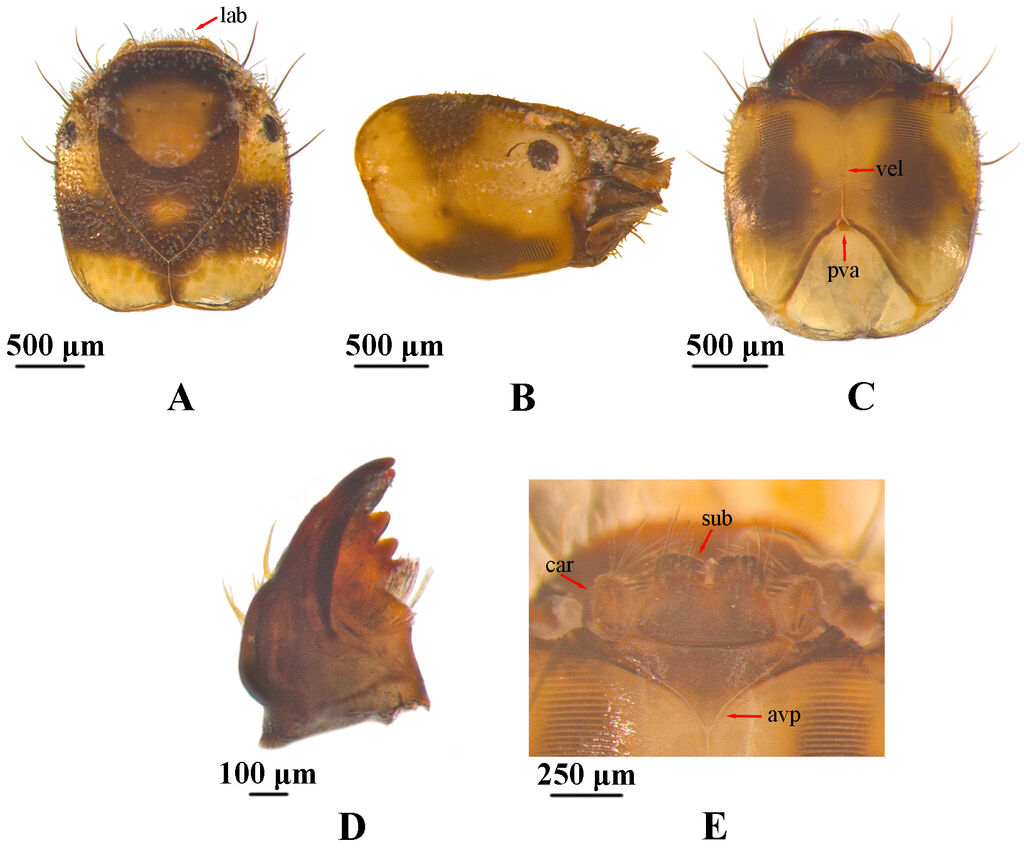
Head of *H.briareus*. **A** dorsal; **B**, right lateral; **C**, ventral; **D**, left mandible, dorsal; **E**, submentum, cardo and anterior ventral apotome, ventral. Abbreviations: avp. anterior ventral apotome; car. cardo; lab. labrum; pva. posterior ventral apotome; sub. submentum; vel. ventral ecdysial line.

**Head.** The head is rectangular in dorsal view, about 1.9 mm long and 1.7 mm wide (Fig. 2A). Dorsum of head uniformly brown and yellowish-brown. Brown is primarily concentrated in the frontoclypeal apotome and both sides of the parietal bone, while there is a yellowish-brown area around the eyes and the posterior margin of parietal. In dorsal view, two yellowish marks are distributed on the frontoclypeal apotome, both presented in a circular shape. The pattern near the anterior margin is larger, shield-shaped, while the pattern near the coronal suture of the head is significantly smaller. Anterior margin of frontoclypeal apotome is nearly straight. Frontoclypeal apotome with posterior angle ogival, at an angle of about 100°. Head in lateral view is nearly trapezoid, with posterolateral regions yellowish, mandible triangular and margin dark brown, eyes oval and black (Fig. 2B). In ventral view, anterior ventral apotome nearly triangular, with anterior border slightly concave and anterolateral angles rounded (Fig. 2C). Ventral ecdysial line nearly twice as long as anterior ventral apotome. Posterior ventral apotome tiny, triangular, brown. Many acuminate peg-like setae and truncate peg-hair setae distributed throughout the dorsum of head, while many long-slender setae only distributed on the front half part of the head. Mandibles dark brown (Fig. 2D), triangular in dorsal view. Left mandible with about five setae at lateral margin and with brush of about a dozen stiff hairs at middle of inner side, with apical tooth and four subapicomesal teeth. Submentum (Fig. 2E) in ventral view brown, with basal 2/3 somewhat trapezoidal and distal 1/3 divided, forming two lobes, lobes nearly square. Submentum posterior margin slightly convex posteriorly, each anterolateral corner with some few stout long setae and many short setae.

**Figure 3.**
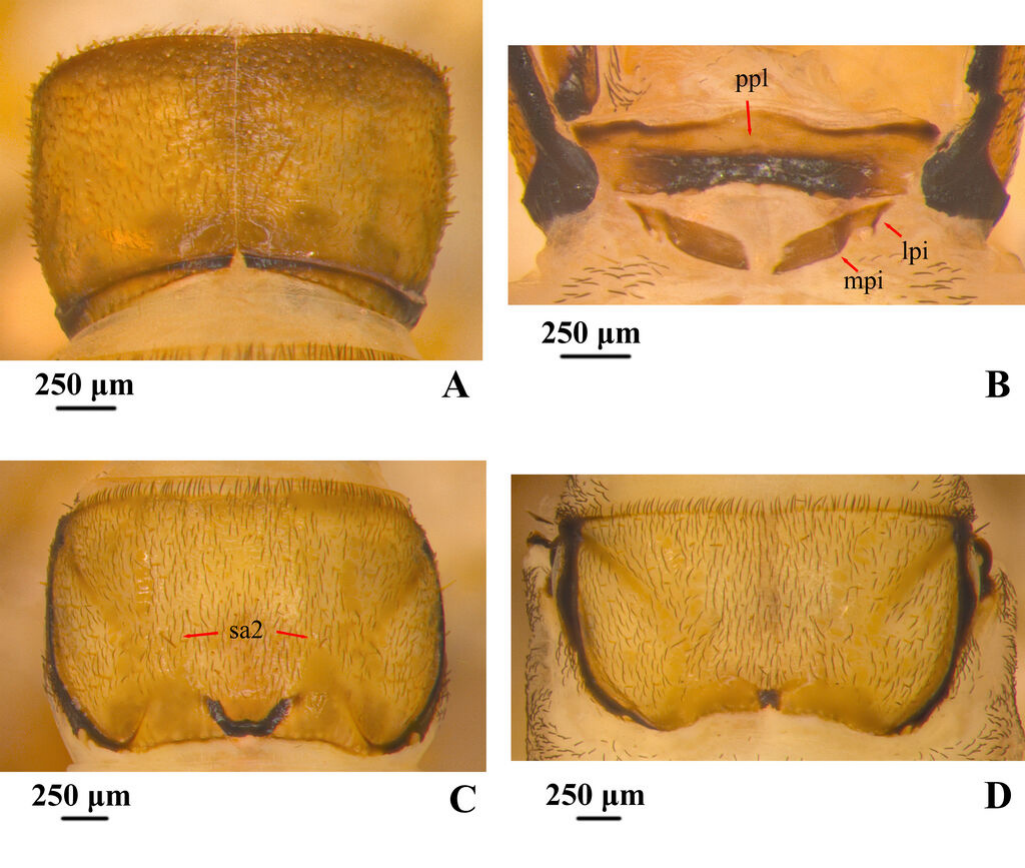
Thorax of *H.briareus*. **A** pronotum, dorsal; **B** prosternal plates and posterior prosternal sclerites, ventral; **C** mesonotum, dorsal; **D** metanotum, dorsal. Abbreviations: lpi. lateral piece; mpi. median piece; ppl. prosternal plate.

**Thorax.** Pronotum (Fig. 3A) subrectangular and yellowish-brown in dorsal view, pronotum subdivided longitudinally by mid-dorsal ecdysial line, both sides are shaped nearly like squares and covered with many short acuminate peg-like setae, hair-like setae and few truncate peg-hair setae. Anterior margin of pronotum straight on both sides of mid-dorsal ecdysial line and colour is darker than the middle; posterior margin shows a circular arc shape in dark brown on both sides of mid-dorsal ecdysial line, concave inward at mid-dorsal ecdysial line, concave-shaped rectangular. Prosternal plate (Fig. 3B) trapezoidal, with width about 4 times its length, slight protrusions on the left and right quarter; anterior margin sinuous, protruding outwards in the middle, the colour is dark on the sides and light in the middle. Posterior margin slightly concave inwards, the dark area in the middle is rectangular. Lateral piece and median piece behind each end of posterior prosternal sclerite fused into rhombic piece on each side, each with anterolateral and posteromesal corners acute, with width about 3.5 times its length. Mesonotum (Fig. 3C) subrectangular and lightly yellowish-brown in dorsal view, covered with dark hair-like setae and truncate peg-hair setae, one long setae at positions sa2. Anterior margin approaching a straight line in brown; posterior margin mark black, concave slightly inward and U-shaped in the middle. Metanotum (Fig. 3D) subrectangular lightly yellowish and length less than mesonotum and pronotum in dorsal view, Broad and deep diagonal groove issuing from each anterolateral angle extending 2/3 distance towards posterior meson of notum. Muscle scars darker than background, longitudinally arranged. Anterior margin of metanotum similar to those of mesonotum; posterior margin shallowly concave, with mesal black quadrate mark. Setae on metanotum and mesonotum less dense than on pronotum. Mesosternum and metasternum with one and two pairs of single-stemmed gill, respectively.

**Figure 4.**
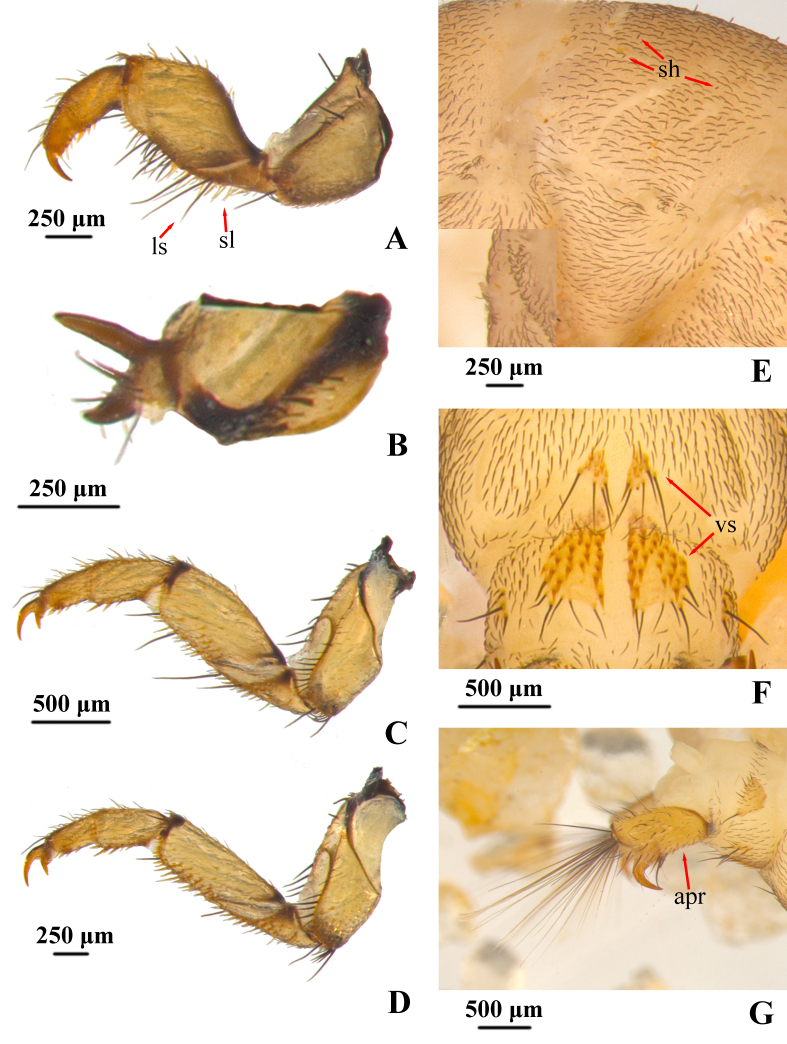
Legs and abdomen of *H.briareus*. **A** left foreleg, left lateral; **B** left propleuron, left lateral; **C** left mid-leg, left lateral; **D** left hind leg, left lateral; **E** setae of abdominal tergum VI, left dorsolateral; **F** plates on abdominal sterna VIII and IX, ventral; **G** anal prolegs, left lateral. Abbreviations: apr. anal proleg; ls. long slender setae; sh. scale-hair setae; sl. spike-like setae; vs. ventral sclerites.

**Legs.** Legs yellowish-brown. Forelegs slightly shorter and thicker than mid- and hind legs (Fig. 4A, 4C and 4D). Each fore-trochantin short and thick bifurcated, upper branch slender, lower one stout, two branches divergent at angle of about 90º, with about 8 setae. Fore coxae shorter and thicker than mid- and hind coxae and shaped conical; mid- and hind coxae are relatively long and shaped cylindrical. Trochanters each two-segmented and approximately triangular, each with basal segment subtriangular and shorter than subtriangular apical segment, ventral margin with more than 15 spike-like setae and two long-slender setae. Fore femora in lateral view pentagonal, each with dorsal margin protruding at mid-length; ventral and dorsal margins with dense long-slender setae and spike-like setae, respectively. Fore tibiae and fore-tarsi tube-like stouter than mid- and hind tibiae and tarsi. Trochanters each two-segmented and approximate triangular with dense spike-like setae and some long slender setae. Mid- and hind femora cylindrical. Tarsal claws of fore-, mid- and hind legs each slightly curved downwards apically.

**Abdomen.** Abdominal sterna with three types of gills: bifid-stemmed gill, single-stemmed gill and pupal gill. Segment I ventrally with two pairs of bifid-stemmed gill, segments II–VI each with ventrolateral bifid-stemmed gill and ventromesal single-stemmed gill, segments III–VI with pupal gill buds laterally, segment VII with pair of bifid-stemmed gills. Abdominal segments I–IX covered densely with black hair-like setae and sparsely with half-erect scale-hair setae (Fig. 4E). There is one long slender seta at each of the sa2 of the abdominal segments I–VII. Subtriangular sterna of segments VIII and IX each with pair of ventral plates (Fig. 4F), yellowish-brown, covered with tapered, short acuminate brown peg-like setae, posterior margin of these sternites with long, black spike-like setae. Anal prolegs and claws yellowish-brown. Anal prolegs (Fig. 4G), each slightly sclerotised with spike-like setae and apical part with about 25 long slender setae arranged in one plane. Anal claws (Fig. 4G) hook-like, angled about 60º.

#### Diagnosis

The larva of *H.briareus* is very similar to the larva of *H.serpentina* Schmid, 1965 in the cephalic colour patterns and the overall shape in dorsal view, but differs from it in the following characteristics: (1) the posterior margin of metanotum has a mesal black punctate mark; (2) frontoclypeal apotome has two yellowish-brown stripes, the anterior margin is larger, shield-shaped; (3) the anterior margin of the frontoclypeal apotome is slightly concave; (4) the posterior angle of frontoclypeal apotome is ogival, at an angle of about 100°, with posterior portion of each frontoclypeal suture slightly sinuous.

### Hydropsyche kozhantschikovi

Martynov, 1924

31E0EE62-768F-5A0C-8937-BD38495C68AB

#### Materials

- **Type status:** Other material. **Occurrence:** sex: 1 male; lifeStage: 1 adult 3 larvae; occurrenceID: CDE781DD-6C19-5330-914A-FCBA86E5D750; **Location:** country: China; stateProvince: Liaoning; county: Benxi Manchu Autonomous county; verbatimLocality: Caohezhang Town, Hujiabaozi River; verbatimDepth: 518 m; verbatimLatitude: 41°7'15" N; verbatimLongitude: 124°14'17" E; verbatimCoordinateSystem: degrees minutes seconds; **Identification:** identifiedBy: Xinyu Ge; **Event:** samplingProtocol: D-frame aquatic net; eventDate: 26 Jul 2023; **Record Level:** institutionCode: Tianjin Normal University, Tianjin, China (TJNU)
- **Type status:** Other material. **Occurrence:** lifeStage: 2 larvae; occurrenceID: D839D75E-7B14-5085-A1AC-9B1DA5AFCD86; **Location:** country: China; stateProvince: Tianjin; county: Jizhou; locality: Baxianshan; verbatimDepth: 293 m; verbatimLatitude: 41°0'41"N; verbatimLongitude: 124°13'14" E; verbatimCoordinateSystem: degrees minutes seconds; **Identification:** identifiedBy: Xinyu Ge; **Event:** samplingProtocol: D-frame aquatic net; eventDate: 2 Jul 2023; **Record Level:** institutionCode: Tianjin Normal University, Tianjin, China (TJNU)
- **Type status:** Other material. **Occurrence:** lifeStage: 10 larvae; occurrenceID: D375F30C-6ECC-58E5-A48A-6776056DD83B; **Location:** country: China; stateProvince: Liaoning; county: Huanren Manchu Autonomous county; locality: Toudao Gou Men, Da Er River; verbatimDepth: 555 m; verbatimLatitude: 41°25'5" N; verbatimLongitude: 124°52'3" E; verbatimCoordinateSystem: degrees minutes seconds; **Identification:** identifiedBy: Xinyu Ge; **Event:** samplingProtocol: D-frame aquatic net; eventDate: 29 Jul 2023; **Record Level:** institutionCode: Tianjin Normal University, Tianjin, China (TJNU)
- **Type status:** Other material. **Occurrence:** sex: 1 male; lifeStage: adult; occurrenceID: E0014630-D255-5051-8DA0-BD1C56799E13; **Location:** country: China; stateProvince: Liaoning; county: Xinbin Manchu Autonomous county; locality: Wudaogou Village, Juliu River; verbatimDepth: 413 m; verbatimLatitude: 41°31'52" N; verbatimLongitude: 125°11'46" E; verbatimCoordinateSystem: degrees minutes seconds; **Identification:** identifiedBy: Xinyu Ge; **Event:** samplingProtocol: UV light trap; eventDate: 29 Jul 2023; **Record Level:** institutionCode: Tianjin Normal University, Tianjin, China (TJNU)
- **Type status:** Other material. **Occurrence:** sex: 1 male; lifeStage: adult; occurrenceID: EB85CDF7-9041-524C-9469-DB7672FE4965; **Location:** country: China; stateProvince: Heilongjiang; county: Human county; locality: Huma River; verbatimDepth: 413 m; verbatimLatitude: 51°39'52" N; verbatimLongitude: 126°36'27" E; verbatimCoordinateSystem: degrees minutes seconds; **Identification:** identifiedBy: Xinyu Ge; **Event:** samplingProtocol: UV light trap; eventDate: 6 Jul 2024; **Record Level:** institutionCode: Tianjin Normal University, Tianjin, China (TJNU)
- **Type status:** Other material. **Occurrence:** sex: 2 males; lifeStage: adult; occurrenceID: 6807AE21-4CED-51B8-9C6B-797444AA7979; **Location:** country: China; stateProvince: Xinjiang Uygur Autonomous Region; county: Fuyun county; locality: Eerqisi River; verbatimDepth: 205 m; verbatimLatitude: 46°59'29" N; verbatimLongitude: 89°33'15" E; verbatimCoordinateSystem: degrees minutes seconds; **Identification:** identifiedBy: Xinyu Ge; **Event:** samplingProtocol: UV light trap; eventDate: 7 Jul 2024; **Record Level:** institutionCode: Tianjin Normal University, Tianjin, China (TJNU)
- **Type status:** Other material. **Occurrence:** sex: 1 male; lifeStage: adult; occurrenceID: C97EB15D-29F9-5FC2-886C-1683B2F385F8; **Location:** country: China; stateProvince: Inner Mongolia Autonomous Region; county: Manzhouli City; locality: Erzi Rivers; verbatimDepth: 205 m; verbatimLatitude: 46°59'29" N; verbatimLongitude: 89°33'15" E; verbatimCoordinateSystem: degrees minutes seconds; **Identification:** identifiedBy: Xinyu Ge; **Event:** samplingProtocol: UV light trap; eventDate: 8 Aug 2024; **Record Level:** institutionCode: Tianjin Normal University, Tianjin, China (TJNU)

#### Description

**Figure 5.**
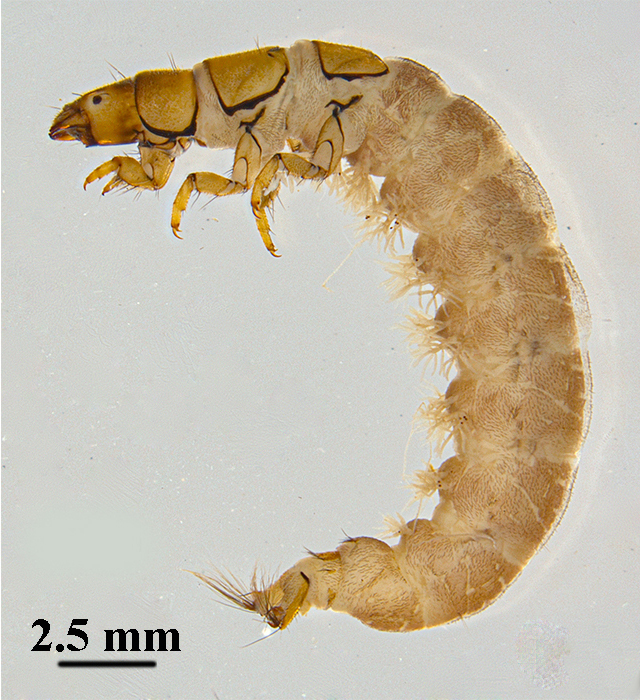
The overall body shape of larval *H.kozhantschikovi* in lateral view.

Mean length of 5^th^ instar larvae about 21 mm (n = 5). Overall body shape as usual in hydropsychid larvae (Fig. 5). The colours of the specimens in alcohol are as follows: body creamy-white; head dark brown with yellow stripe; pronotum, ventral prosternum, abdominal sternum, legs and anal claws yellowish-brown.

**Figure 6.**
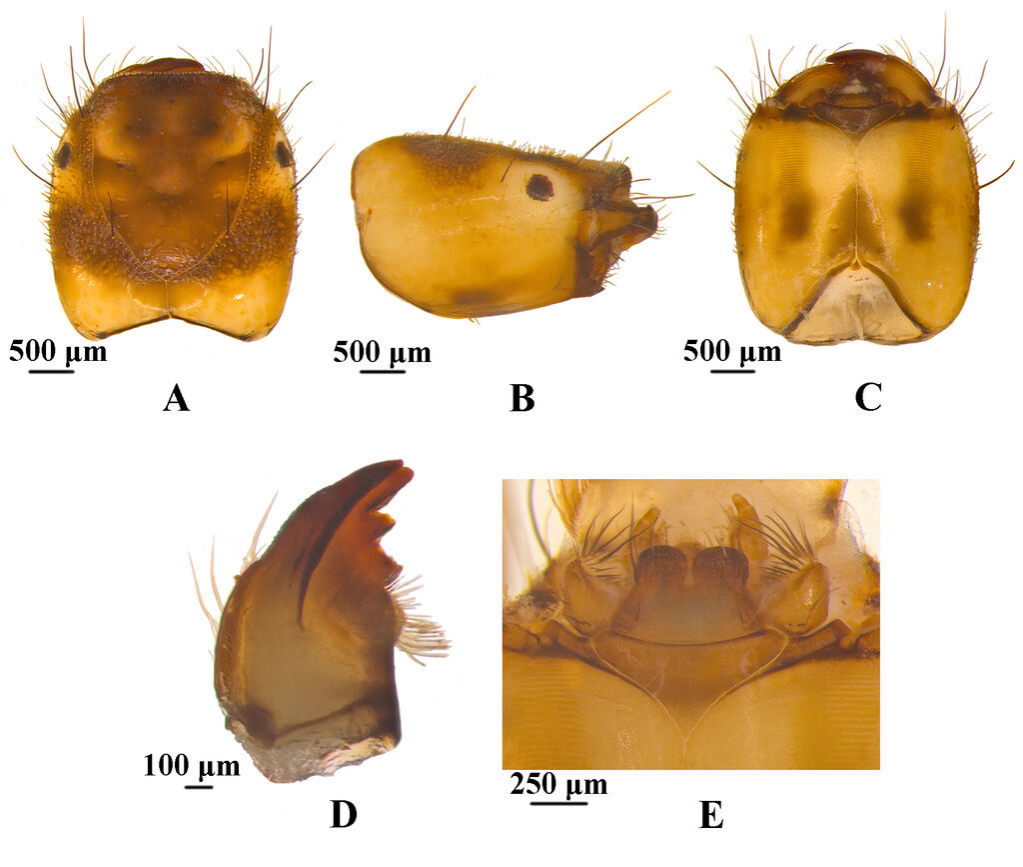
Head of *H.kozhantschikovi*. **A** dorsal; **B** right lateral; **C** ventral; **D** left mandible, dorsal; **E** submentum, cardo and anterior ventral apotome, ventral.

**Head.** Head at dorsal view roughly rectangular, about 2.7 mm long and 2.4 mm wide. Dorsum of head mostly light brown, with yellowish area around eyes, frontoclypeal apotome and parietal (Fig. 6A). Yellowish marks divided into three columns with two marks in each column on the frontoclypeal apotome, the stripes on the front left and front right are crescent-shaped, with rest of the marks nearly circular. Anterior margin of frontoclypeal apotome slightly concave, with mediotransversal fold arm conspicuous, anterior margin slightly convex, posterior angle ogival, about 120°. Dorsum of head with dark brown hair-like setae, short and brown truncate peg-like setae and short acuminate peg-like setae. Head in lateral view nearly trapezoid, parietal almost yellowish, eyes oval and black (Fig. 6B). In ventral view (Fig. 6C), pair of files brown, parietals otherwise yellow; anterior ventral apotome nearly triangular, brown, with anterior border slightly concave and anterolateral angles rounded. Ventral ecdysial line about 3 times the length of anterior ventral apotome. Posterior ventral apotome tiny, triangular, brown. There is a concave-shaped triangle at 2/3 distance posterior of ventral ecdysial line. Mandibles (Fig. 6D) triangular, dark yellow basally and mostly brown in ventral view, each with two apical teeth and 3 subapicomesal teeth; left mandible with about 10 setae at lateral margin and with brush of about dozen stiff hairs at middle of inner side. Right and left mandibles nearly symmetrical. Submentum (Fig. 6E) in ventral view with basal 2/3 somewhat trapezoidal and distal 1/3 divided, forming two lobes, two lobes nearly oval, posterior margin slightly convex posteriorly. Each anterolateral corner with few long setae and many short setae and each posterolateral corner with stout, short setae. Maxilla (Fig. 6E) each with cardo drop-shaped, brown, stipes.

**Figure 7.**
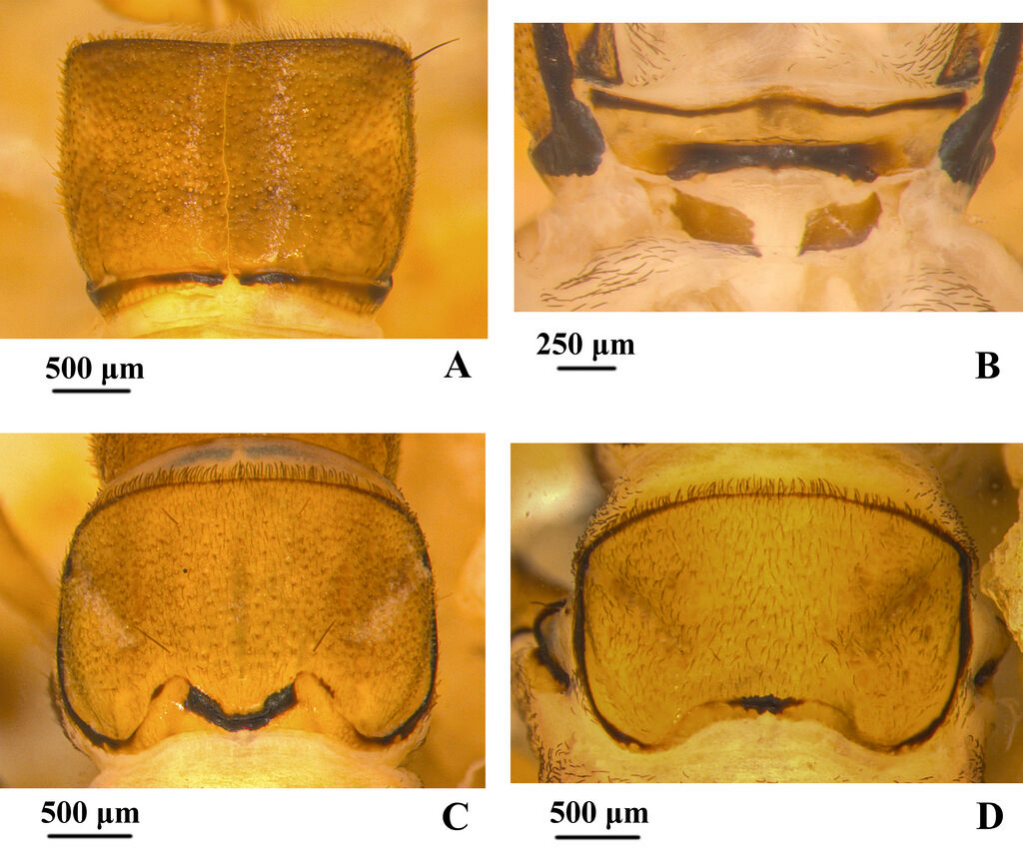
Thorax of *H.kozhantschikovi* Martynov, 1924. **A** pronotum, dorsal; **B** prosternal plates and posterior prosternal sclerites, ventral; **C** mesonotum, dorsal; **D** metanotum, dorsal.

**Thorax.** Pronotum (Fig. 7A) subrectangular and yellowish-brown, with its width about 4 times its length in dorsal view, subdivided by mid-dorsal ecdysial line. Anterior margin straight and dark brown, posterior margin slightly concave black or dark brown. Pronotum covered with many brown short acuminate peg-like setae, hair-like setae and few truncate peg-hair setae in dorsal view. Prosternal plate large (Fig. 7B), nearly trapezoidal, with its width about 5 times its length; anterior margin blackish, slightly sinuous and slightly convex anteriorly on meson, posterior margin slightly concave. Intersegmental fold often covering two pairs of sclerites posterior of prosternal sclerite; on each side, lateral and submesal sclerite pieces behind prosternal sclerite fused into subrhombic sclerites (Fig. 7B) and with width about 3.5 times its length. Mesonotum (Fig. 7C) yellow brown in dorsal view, undivided on mid-line, with anterior margin straight, anterolateral angles and lateral margins and posterolateral angles black, pair of tint lines issuing from anterolateral angles extending anteromesad to level of ends of V-shaped black mark on middle of posterior margin, each line with anteromesal end bulging and darkened. Diagonal grooves indistinct. Anterior margin with many black hair-like setae and few truncate or acuminate peg-like setae. Metanotum (Fig. 7D) subrectangular and lighter than pronotum and mesonota in lightly yellowish-brown and undivided on mid-line in dorsal view. Anterior margin slightly sinuous, convex anteriorly on meson. Posterior margin shallowly concave, slightly sinuous black mark on middle of posterior margin, anterolateral angles and lateral margins black; broad and deep diagonal grooves issuing from anterolateral angles and more conspicuous than those of mesonotum extending 2/3 distance towards posterior meson of notum. Muscle scars darker than background, longitudinally arranged. Setae on mesonotum and metanotum less dense than those on pronotum. Mesosternum and metasternum with one and two pairs of single-stemmed ventral gills, respectively.

**Figure 8.**
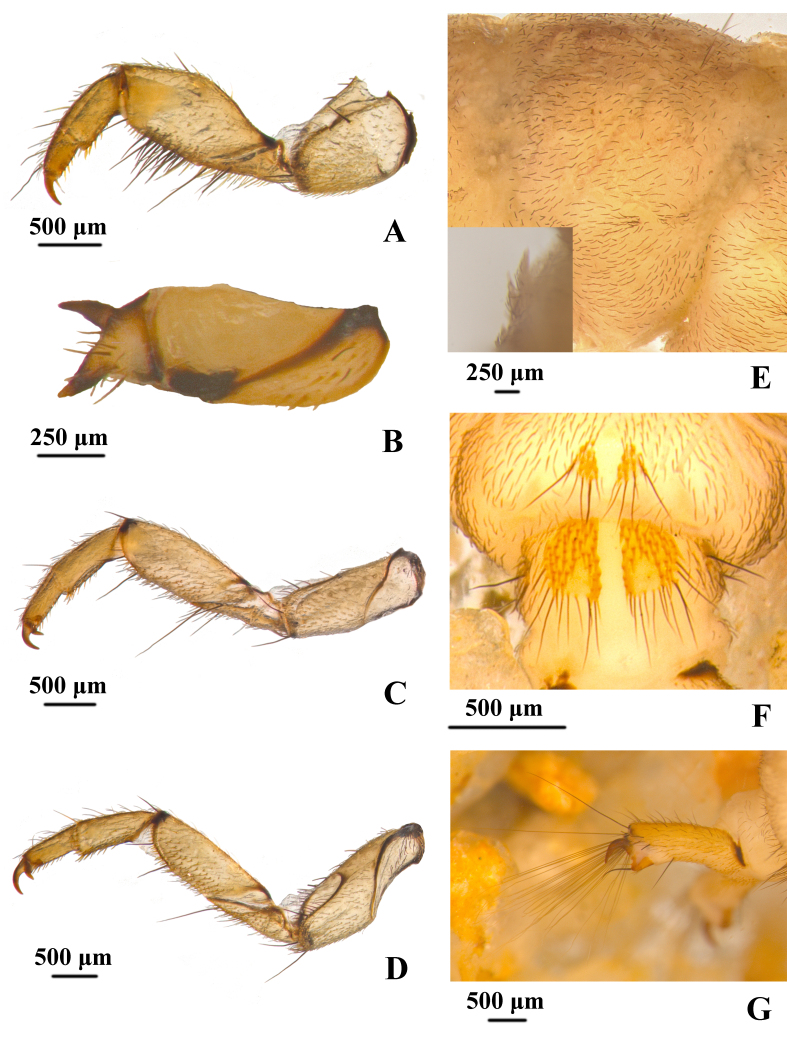
Legs and abdomen of *H.kozhantschikovi*. **A** right foreleg, right lateral; **B** right propleuron, right lateral; **C** right mid-leg, right lateral; **D** right hind-leg, right lateral; **E** setae of abdominal tergum VI, left dorsolateral; **F** plates on abdominal sterna VIII and IX, ventral; **G** anal prolegs, left lateral.

**Legs.** Legs yellowish-brown. Forelegs slightly shorter and thicker in structure than mid- and hind legs (Fig. 8A, 7C and 7D). Each fore-trochantin bifurcate (Fig. 8B), two branches divergent at angle of about 90º, with about 10 setae, upper branch slender, lower one stout. Fore-coxae shorter than mid- and hind coxae and somewhat conical, mid- and hind coxae are relatively long and shaped cylindrical in lateral view. Trochanters (Fig. 8B) each two-segmented and approximate triangular, each with basal segment subtriangular and shorter than subtriangular apical segment, trochanteral brush usually present on apical segment, ventral margin with more than 20 spike-like setae and two-three long slender setae. Fore-femora in lateral view pentagonal, dorsal and ventral margins with dense long slender setae and spike-like setae, respectively. Fore-tibiae and fore-tarsi shorter and stouter than mid- and hind tibiae and tarsi. Mid- and hind femora cylindrical, with few long slender setae and several shorter spike-like setae. Trochanters each two-segmented and approximately triangular, with dense spike-like setae and some long slender setae. Every tibiae and tarsus shaped like tube, forelegs are stouter than those of mid- and hind legs. Tarsal claws slightly curved downwards, of which mid-legs and hind legs fork.

**Abdomen.** Abdominal segments I–IX covered densely with slender, dark hair-like setae, segments I–VIII also covered with flat end, brown scale-hair setae (Fig. 8E). There is one long slender seta at each of the sa2 of the abdominal segments I–VII. Abdominal sterna with three types of gills: bifid-stemmed gill, single-stemmed gill and pupal gill. The gill distribution pattern is similar to that of *H.briareus*. Subtriangular sterna of segments VIII and IX each with pair of ventral plates (Fig. 8F). Pair of sternites VIII subtriangular, yellowish-brown, covered with acuminate peg-like setae; posterior margin of these sternites with long, dark brown spike-like setae. Pair of sternites IX subtrapezoid, yellowish-brown; acuminate peg-like setae on posterior parts of these sternites longer and thicker than those of segment VIII; posterior margin also covered with long, dark brown spike-like setae. Anal prolegs and claws yellowish-brown (Fig. 8G). Anal claws hook-like, angled about 60º, basal part of anal prolegs with about 30 long slender setae.

#### Diagnosis

The larva of *H.kozhantschikovi* can be diagnosed by the combination of the following features: (1) frontoclypeal apotome with posterior angle ogival, about 120°, with posterior portion of each frontoclypeal suture slightly sinuous; (2) frontoclypeal apotome have yellow stripes divided into three columns with two patches in each column on the frontoclypeal apotome, the stripes on the front left and front right being crescent-shaped, while the rest are circular; (3) each fore-trochantin bifurcate, two branches divergent at angle of about 90º.

## Identification Keys

### Key to 5^th^ instar larvae of 21 Chinese species of *Hydropsyche*.

**Table:** 

1	Dorsum of head uniformly black or blackish brown, except for small pale areas around eyes (Zhou 2007, fig. 24a; Ge et al. 2020, fig.2A)	2
–	Dorsum of head with marks or stripes (Figs 5A, 8A)	4
2	Head capsule dorsum with posterolateral corners black to brown (Zhou 2007, fig.24a)	*H.grahami* Banks, 1940 (morphotype cl)
–	Head capsule dorsum with posterolateral corners of parietals light yellow	3
3	Anterior margin of frontoclypeal apotome slightly concave (Hur et al. 2000, fig. 1)	*H.orientalis* Martynov, 1934
–	Anterior margin of frontoclypeal apotome straight (Ge et al. 2020, fig. 2A)	*H.cerva* Li & Tian, 1990
4	Anterior margin of frontoclypeal apotome with upturned tooth or denticle on each side (Lepneva 1964, fig. 678; Zhou 2007, fig. 28a)	5
–	Anterior margin of frontoclypeal apotome straight or slightly concave, without teeth (Xu et al. 2018, figs. 3A, 6A, 9A and 12A; Ge et al. 2020, figs. 5A and 8A)	6
5	Anterior margin of frontoclypeal apotome symmetrical (Lepneva 1964, fig. 678)	*H.ornatula* McLachlan, 1878
–	Anterior margin of frontoclypeal apotome asymmetrical, more convex on right side than on left side (Zhou 2007, fig. 28a)	*H.quadrata* (Li & Dudgeon, 1990)
6	Frontoclypeal apotome without marks, stripes or spots, but with paired longitudinal stripes along frontoclypeal sutures from base to anterior ends (Zhou 2007, fig. 25a; Xu et al. 2018, fig. 3A)	7
–	Frontoclypeal apotome with diverse marks, stripes or spots; without longitudinal stripes along frontoclypeal sutures from base to anterior ends (Lepneva 1964, fig. 678; Xu et al. 2018, figs. 6A, 9A and 12A; Ge et al. 2020, figs. 5A and 8A)	8
7	Metanotum with distinct longitudinal stripes (Zhou 2007, figs. 25a and 25c)	*H.formosana* Ulmer, 1911
–	Metanotum yellow, without stripes (Xu et al. 2018, fig. 3A)	*H.arion* Malicky & Chantaramongkol, 2000
8	Triangular anterior ventral apotome nearly isosceles; anterior margin of ventral ecdysial line with shallow notch (Fischer and Neu 2002, figs. 2j and l)	9
–	Triangular anterior ventral apotome nearly equilateral; anterior margin of ventral ecdysial line with deep notch (Xu et al. 2018, figs. 6B, 9B and 12B; Ge et al. 2020, figs. 8E and 5E)	10
9	Centre of black spots (pretentorinae) on frontoclypeal apotome expressed as ratio of distance between the pretentorinae/sum of distances between pretentorinae and lateral clypeus margin (1.31–1.74) (Fischer and Neu 2002, fig. 2c)	*H.botosaneanui* Marinković-Gospodnetić, 1966
–	Centre of black spots (pretentorinae) on frontoclypeal apotome expressed as ratio of distance between pretentorinae/sum of distances between pretentorinae and lateral clypeus margin 2.26–2.52) (Fischer and Neu 2002, fig. 2a)	*H.pellucidula* (Curtis, 1834)
10	Dark marks on frontoclypeal apotome A-shaped from base to mediotransversal folding (Zhou 2007, figs. 21a and 22a)	11
–	Dark marks on frontoclypeal apotome not A-shaped, but with oval marks (Xu et al. 2018, figs. 6A, 9A and 12A) or more than two pairs of stripes (Zhou 2007, fig. 26a)	13
11	Centre of frontoclypeal apotome with numerous truncate peg-like setae (Zhou 2007, fig. 21a)	*H.grahami* Banks, 1940 (morphotype c10)
–	Centre of frontoclypeal apotome without peg-like setae (Zhou 2007, fig. 22a; Ge et al. 2020, fig. 8A)	12
12	Frontoclypeal apotome with anterolateral corners not appearing anterolaterad (Zhou 2007, fig. 22a)	*H.furcula* Tian & Li, 1985
–	Frontoclypeal apotome with anterolateral corners appearing anterolaterad (Ge et al. 2020, fig. 8A)	*H.uvana* Mey, 1995
13	Anterior margin of submentum with shallow notch, much shorter than half of mesal line of basal submentum (Zhou 2007, fig. 26b)	*H.polyacantha* Li & Tian, 1989
–	Anterior margin of submentum with deep notch, at least half as long as mesal line of basal submentum (Ge et al. 2020, figs. 6E, 9E and 12E)	14
14	Trochantins each with two branches divergent less than 90º (Xu et al. 2018, fig. 13D; Ge et al. 2020, fig. 7B)	15
–	Trochantins each with two branches divergent about 90º (Xu et al. 2018, figs. 7D and 10D)	17
15	Prosternal plate with its width about 6 times its length (Xu et al. 2018, fig. 13E)	*H.trifora* (Li & Tian, 1990)
–	Prosternal plate with its width about 4 times its length (Zhou 2007, fig. 19f)	16
16	Submesal sclerite piece arc-shaped anteromedially	*H.fukienensis* Schmid, 1965
–	Submesal sclerite piece with anteromedial corners at angle of more than 90º	*H.penicillata* Martynov, 1931
17	Frontoclypeal apotome with 4–9 light marks (Lepneva 1964, fig. 703)	18
–	Frontoclypeal apotome with fewer marks (Xu et al. 2018, figs. 6A and 9A)	19
18	The anteromedian mark longitudinal, oblong, narrower in the middle, while the rest oval (Lepneva 1964, fig. 703)	*H.newae* Kolenati, 1858
–	The marks on the front left and front right are crescent-shaped, while the rest oval (Fig. 6A)	*H.kozhantschikovi* Martynov, 1924
19	Pronotum with two dark brown stripes and two yellow transverse stripes (Xu et al. 2018, fig. 10A)	*H.simulata* Mosely, 1942
–	Pronotum yellow or dark brown, without stripes or marks (Xu et al. 2018, fig. 7A)	20
20	Frontoclypeal apotome with three longitudinal oval marks (Xu et al. 2018, fig. 6A)	*H.columnata* Martynov, 1931
–	Frontoclypeal apotome with two prominent marks (Zhou 2007, fig. 20a)	21
21	Posterior margin of metanotum with mesal black transverse mark	*H.serpentina* Schmid, 1965
–	Posterior margin of metanotum with mesal black punctate mark (Fig. 2A)	*H.briareus* Malicky & Chantaramongkol, 2000

## Discussion

### Classification and biogeography

**Figure 9.**
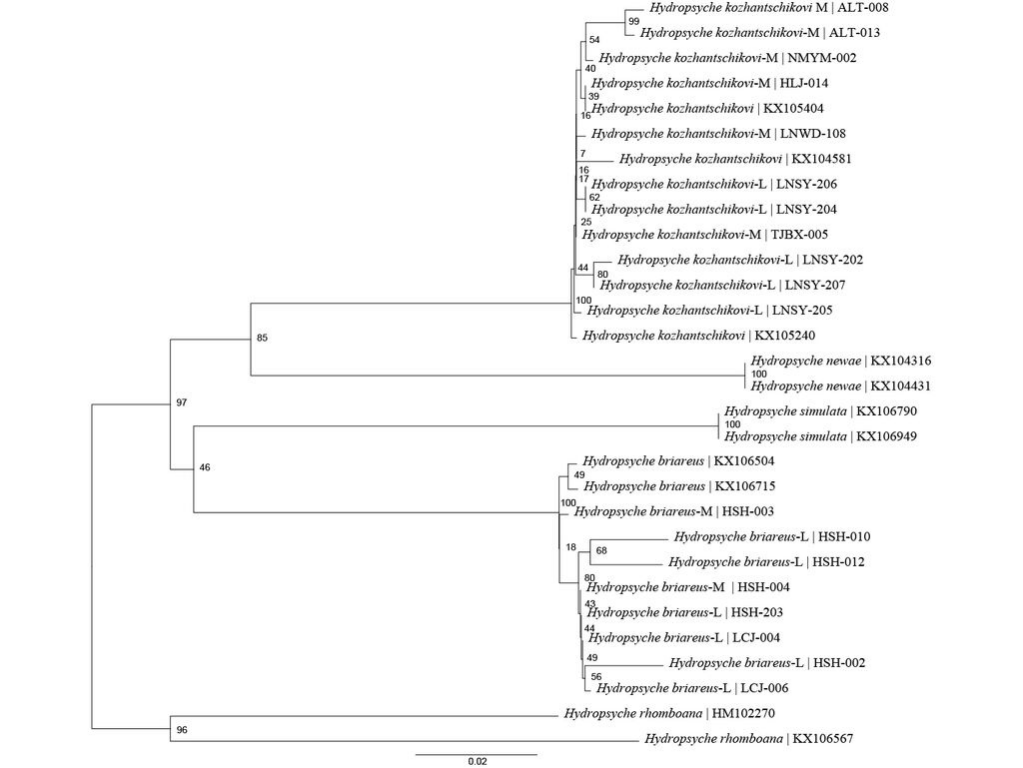
Neighbour-joining tree based on the 658-bp *mtCOI* gene of five *Hydropsyche* species. Numbers on branches represent bootstrap support based on 1000 replicates; scale equals K2P genetic distance.

In this study, we collected adult and larval specimens of both species from parts of northeast, north and southwest China. The molecular identification and morphological taxonomy results align (Fig. 9), suggesting that DNA barcodes and traditional morphological taxonomy complement each other, with the former serving as a straightforward means to enhance the latter's effectiveness. *Hydropsychebriareus* and *H.kozhantschikovi* belong to the *H.newae* species group. This species group includes four clades: *H.newae* species clade, *H.columnata* species clade, *H.simulata* species clade and *H.serpentine* species clade. *H.briareus* and *H.kozhantschikovi* belong to the *H.simulata* species and *H.newae* species clade, respectively. This is consistent with the results of the NJ tree (Fig. 9). According to the new key, we found that the larval characteristics within the species group were also similar.

In addition, we found distinct distribution patterns between *H.kozhantschikovi* and *H.briareus*. The former exhibits a strict Palaearctic distribution, while the latter is endemic to the Oriental realm. Notably, our field surveys have extended the known southern range limit of *H.kozhantschikovi* in China to Tianjin City (Yang et al. 2016), demonstrating a broader latitudinal distribution than previously documented. In contrast, since *H.briareus* was first recorded in China (Ge et al. 2023), its main discovery sites have been confined to the Jinsha River Basin in China (24°–36° N, 90°–105° E). This limited distribution may reflect ecological constraints imposed by the unique microclimatic regime of Basin. These updated biogeographic records provide critical baseline data for modelling species range dynamics under climate change scenarios.

In summary, this study enriches the larval database of Chinese Trichoptera. However, numerous hydropsychid larvae remain undescribed in China. Therefore, future collections should encompass broader geographical areas across the country to facilitate comprehensive taxonomic investigations.

## Supplementary Material

XML Treatment for Hydropsyche briareus

XML Treatment for Hydropsyche kozhantschikovi

3B3027E3-AFD0-53FA-8C9A-C52078749ADF 10.3897/BDJ.13.e151321.suppl1 Supplementary material 1 *mtCOI* of specimens used in larva-male associations Data type phylogenetic Brief description Table S1. *mtCOI* of specimens used in larva-male associations of *Hydropsyche*. File: oo_1270847.docx https://binary.pensoft.net/file/1270847 Xinyu Ge

## Figures and Tables

**Table 1.** PCR primers used to sequence *mtCOI* genes of *Hydropsyche* specimens in this study.

Primer	Sequence	Reference
LCO1490	GGTCAACAAATCATAAAGATATTGG	Folmer et al. (1994)
HCO2198	TAAACTTCAGGGTGACCAAAAAATCA	Folmer et al. (1994)
