# Supplementary material for: Descriptions of hitherto unknown larvae of the genus Hydropsyche Pictet, 1834 from China (Trichoptera, Hydropsychidae)
Source: Biodivers Data J. 2025 Mar 17;13:e151321. doi: 10.3897/BDJ.13.e151321 (PMC11933909; doi:10.3897/BDJ.13.e151321)
Supplement: Supplementary material 1 — mtCOI of specimens used in larva-male associations [file bdj-13-e151321-s001.docx]

**Table. S1 *mtCOI* of specimens used in larva-male associations of *Hydropsyche***

| Sample ID | Species | GenBank ID |
| --- | --- | --- |
| ALT-008 | *H. kozhantschikovi* | PV174544 |
| ALT-013 | *H. kozhantschikovi* | PV174545 |
| NMYM-002 | *H. kozhantschikovi* | PV174548 |
| HLJ-014 | *H. kozhantschikovi* | PV174551 |
| LNSY-205 | *H. kozhantschikovi* | PV174551 |
| LNWD-108 | *H. kozhantschikovi* | PV174547 |
| LNSY-202 | *H. kozhantschikovi* | PV174551 |
| LNSY-207 | *H. kozhantschikovi* | PV174551 |
| LNSY-206 | *H. kozhantschikovi* | PV174551 |
| LNSY-204 | *H. kozhantschikovi* | PV174554 |
| TJBX-005 | *H. kozhantschikovi* | PV174554 |
|  | *H. kozhantschikovi* | KX105404 |
|  | *H. kozhantschikovi* | KX104581 |
|  | *H. kozhantschikovi* | KX105240 |
| HSH-003 | *H. briareus* | PV174554 |
| HSH-010 | *H. briareus* | PV174559 |
| HSH-012 | *H. briareus* | PV174560 |
| HSH-004 | *H. briareus* | PV174555 |
| HSH-203 | *H. briareus* | PV174558 |
| LCJ-004 | *H. briareus* | PV174561 |
| HSH-002 | *H. briareus* | PV174557 |
| LCJ-006 | *H. briareus* | PV174562 |
|  | *H. newae* | KX104316 |
|  | *H. newae* | KX104431 |
|  | *H. briareus* | KX106504 |
|  | *H. briareus* | KX106715 |
|  | *H. simulata* | KX106949 |
|  | *H. simulata* | KX106790 |
|  | *H. rhomboana* | HM102270 |
|  | *H. rhomboana* | KX106567 |
